# Supplementary material for: ProNA3D: Distance-Based Analysis of Nucleic Acid-Containing Interfaces
Source: Comput Struct Biotechnol J. 2026 Sep 14;35(1):0202. doi: 10.34133/csbj.0202 (PMC13618712; doi:10.34133/csbj.0202)
Supplement: Supplementary file 1 [file csbj.0202.f1.zip › revised_Supplemental.pdf]

Supplemental material

Supplemental figures

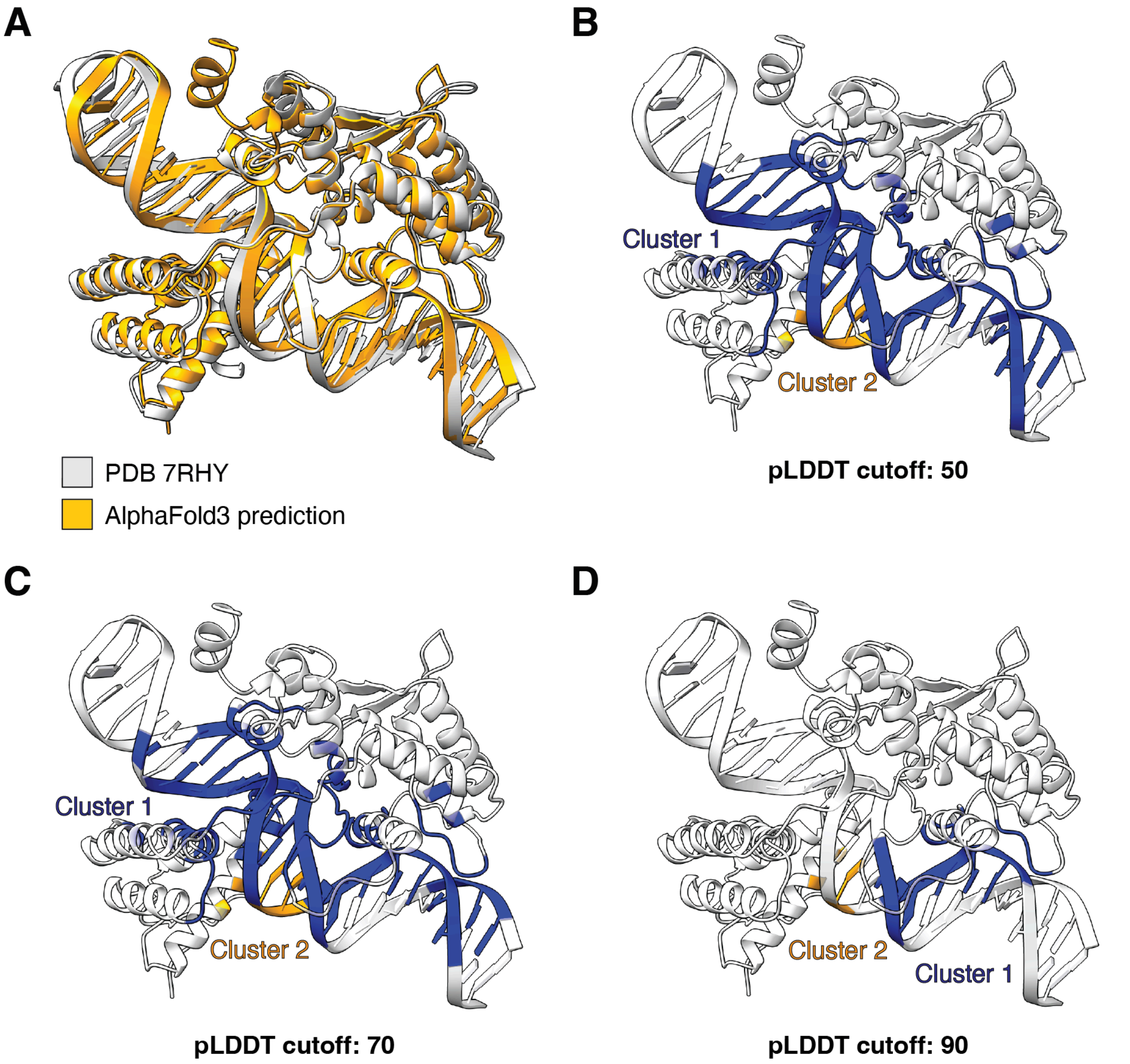

Suppl. Figure 1: ProNA3D analysis of the AlphaFold3-predicted complex of the Cre recombinase from *Punavirus P1* bound to its target DNA. **(A)** Superposition of the AlphaFold3 model (orange, Cre recombinase residues: 1-343, DNA nucleotides: 1-49) on the experimentally determined structure (PDB ID: 7RHY) (white, Cre recombinase residues: 20-329 with unresolved regions not modeled, DNA nucleotides: 1-49). **(B)** Sub-interfaces identified by ProNA3D using a pLDDT cutoff of 50, with sub-interface 1 (105 interacting residues and nucleotides) shown in blue and sub-interface 2 (5 interacting residues and nucleotides) in orange. **(C)** Sub-interfaces detected using a pLDDT cutoff of 70, colored as in (B) (Sub-interface 1: 95 interacting residues and nucleotides, Sub-interface 2: 5 interacting residues and nucleotides). **(D)** Sub-interfaces detected using a pLDDT cutoff of 90, with

sub-interface coloring consistent with (B) (Sub-interface 1: 16 interacting residues and nucleotides, sub-interface 2: 3 interacting residues and nucleotides).

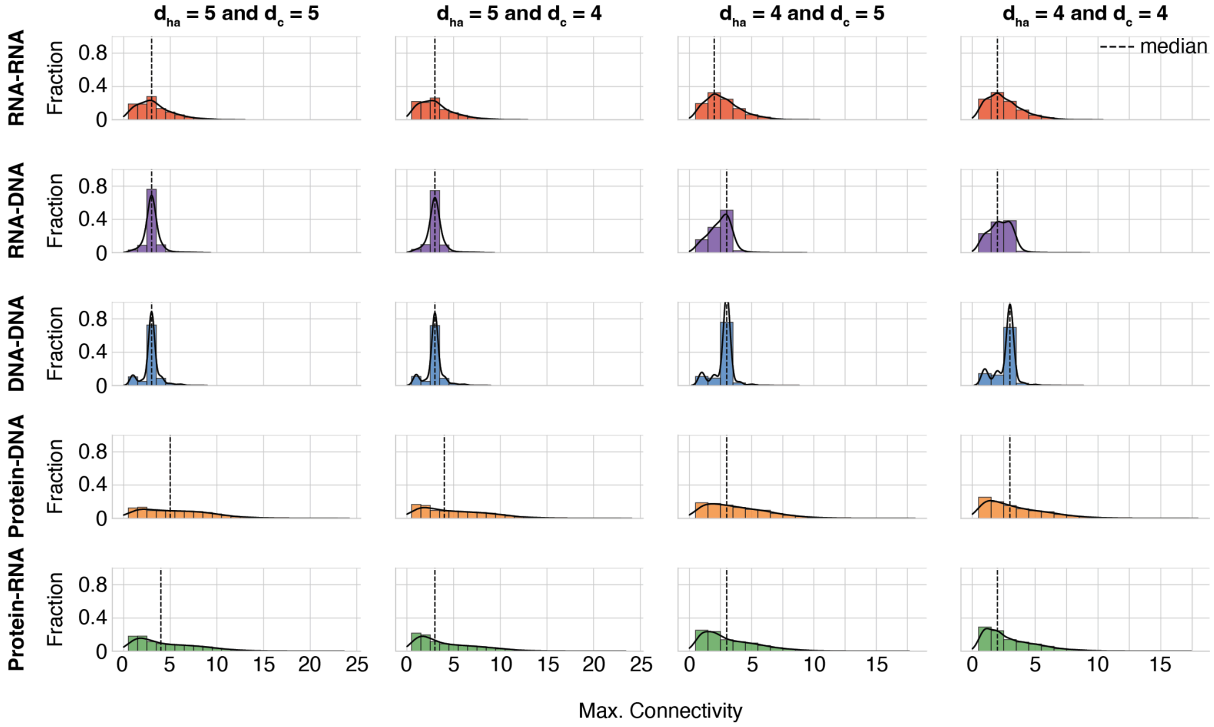

Suppl. Figure 2: Maximum connectivity across nucleic acid-containing interface types and varying d_{HA} and d_C thresholds. Connectivity was defined as the number of distinct residues or nucleotides contacted by a residue or nucleotide across an interface. For each interface, the highest connectivity value was extracted. Shown are RNA-RNA (red), RNA-DNA (purple), DNA-DNA (blue), protein-DNA (orange), and protein-RNA (green) interfaces. Median values are indicated for each interface class (dotted line). **(A)** d_{HA} = 5 and d_C = 5, **(B)** d_{HA} = 5 and d_C = 4, **(C)** d_{HA} = 4 and d_C = 5, **(D)** d_{HA} = 4 and d_C = 4.

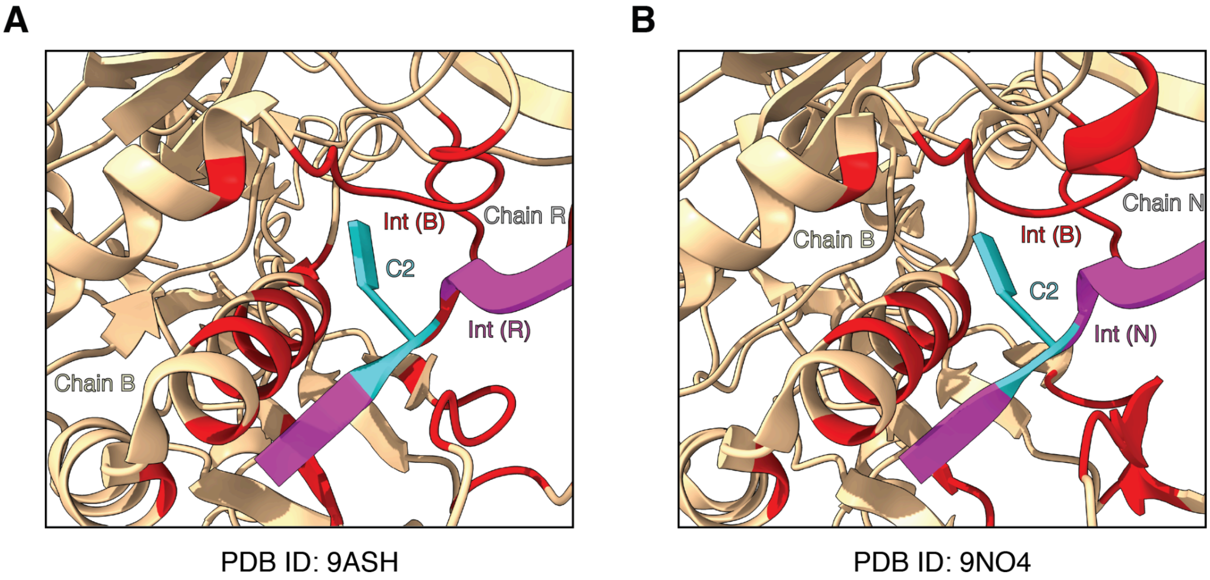

Suppl. Figure 3: Conserved high-connectivity nucleotide flipping in CRISPR-associated protein Csm4 complexes (red: Csm4 interface residues, magenta: RNA interface nucleotides, cyan: flipped high-connectivity nucleotide). **(A)** CRISPR-associated protein Csm4 from *L. lactis* bound to crRNA (PDB: 9ASH). **(B)** CRISPR-associated protein Csm4 from *S. thermophilus*bound to crRNA (PDB: 9NO4).

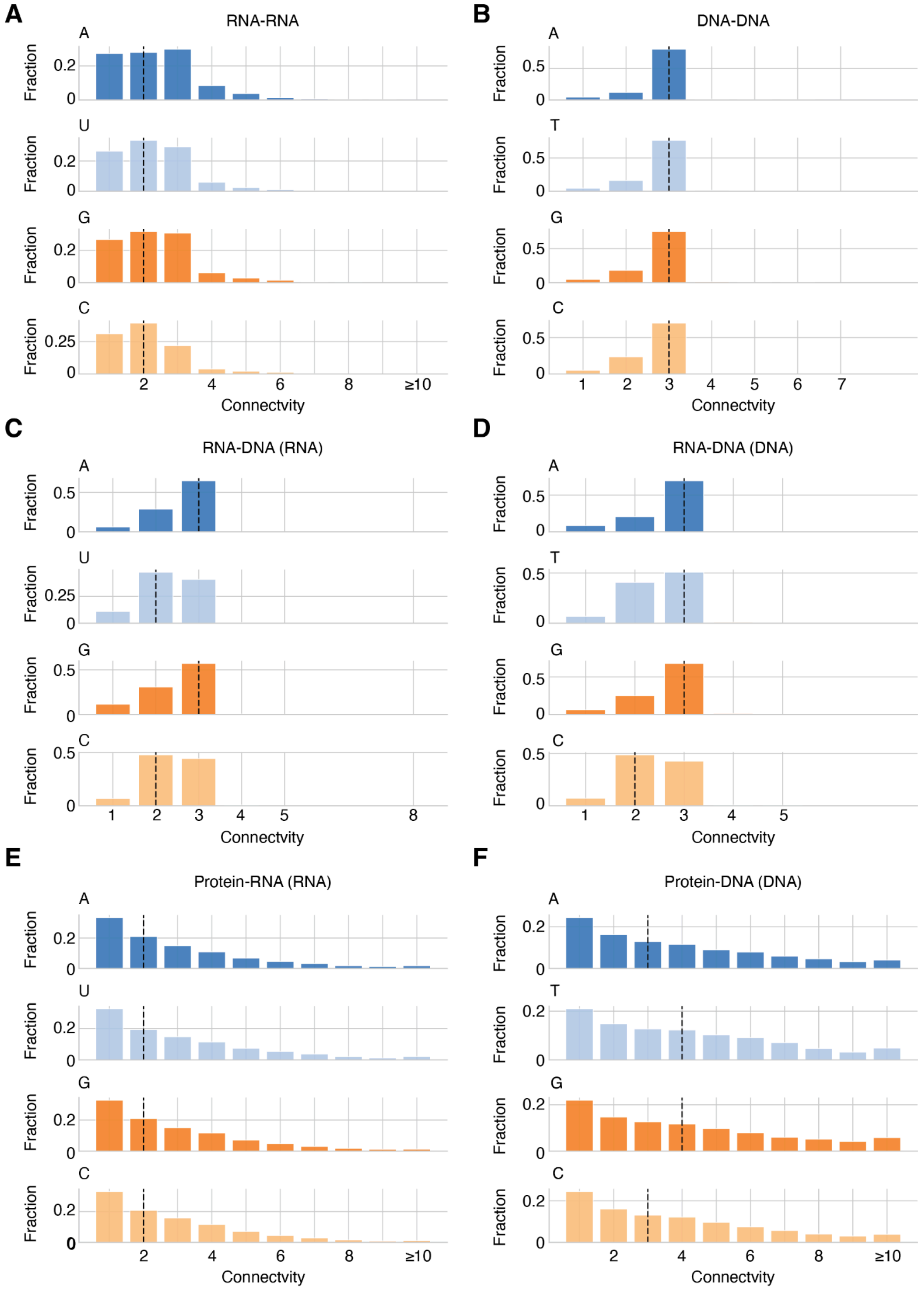

Suppl. Figure 4: Per-nucleotide type connectivity (A/DA: blue; U/DT: lightblue; DG/G: orange; C/DC: lightorange) across nucleic acid-containing interface types. Connectivity was defined as the number of distinct residues or nucleotides contacted by a residue or nucleotide across an interface. **(A)** RNA-RNA, **(B)** DNA-DNA, **(C)** RNA-DNA (RNA), **(D)** RNA-DNA (DNA), **(E)** protein-RNA (RNA), and **(F)** protein-DNA (DNA). Median values are indicated for each interface class (dotted line).

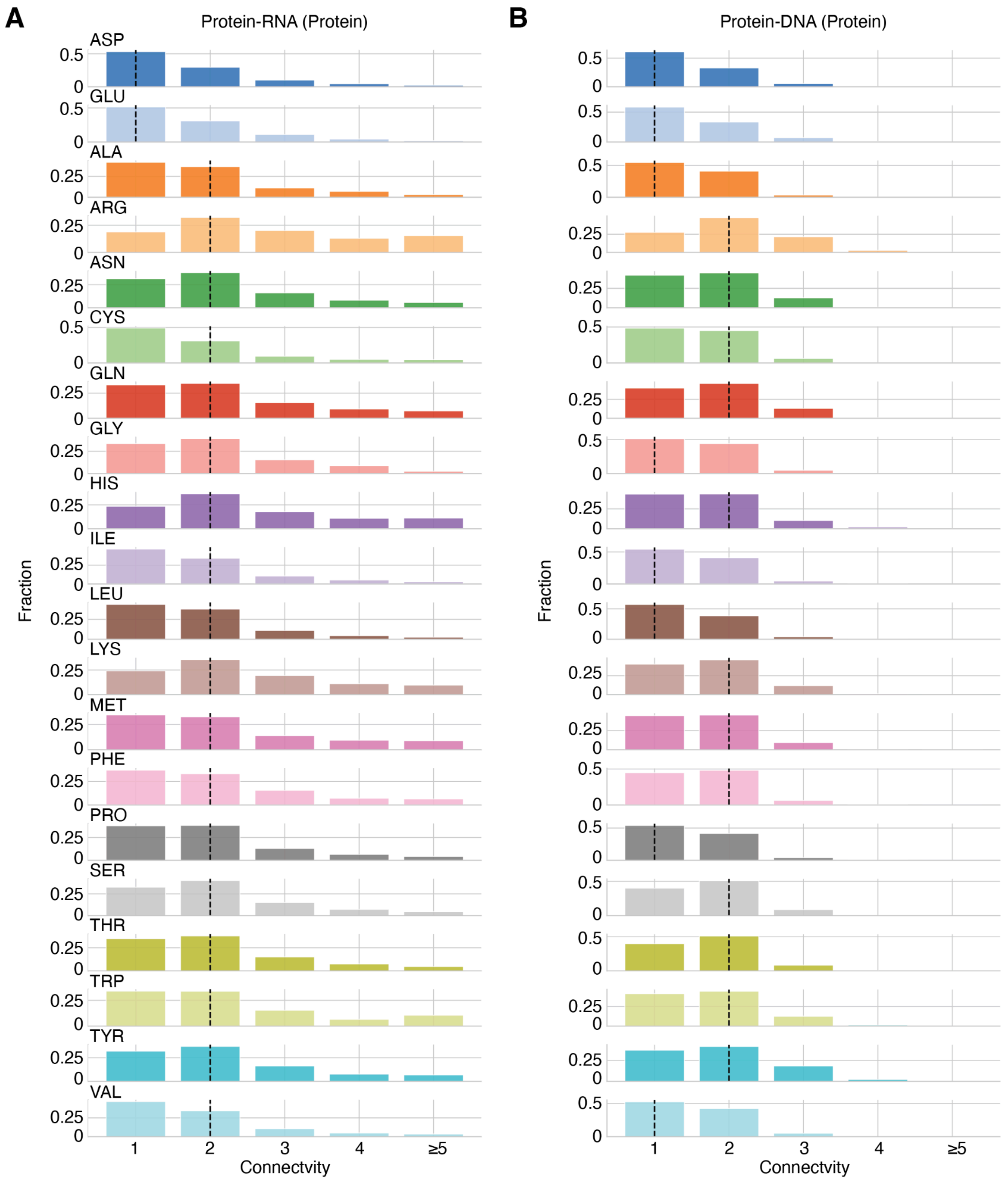

Suppl. Figure 5: Per-residue type connectivity across nucleic acid-containing interface types. Connectivity was defined as the number of distinct residues or nucleotides contacted by a residue or nucleotide across an interface. **(A)** protein-RNA (protein) and **(B)** protein-DNA (protein). Median values are indicated for each interface class (dotted line).

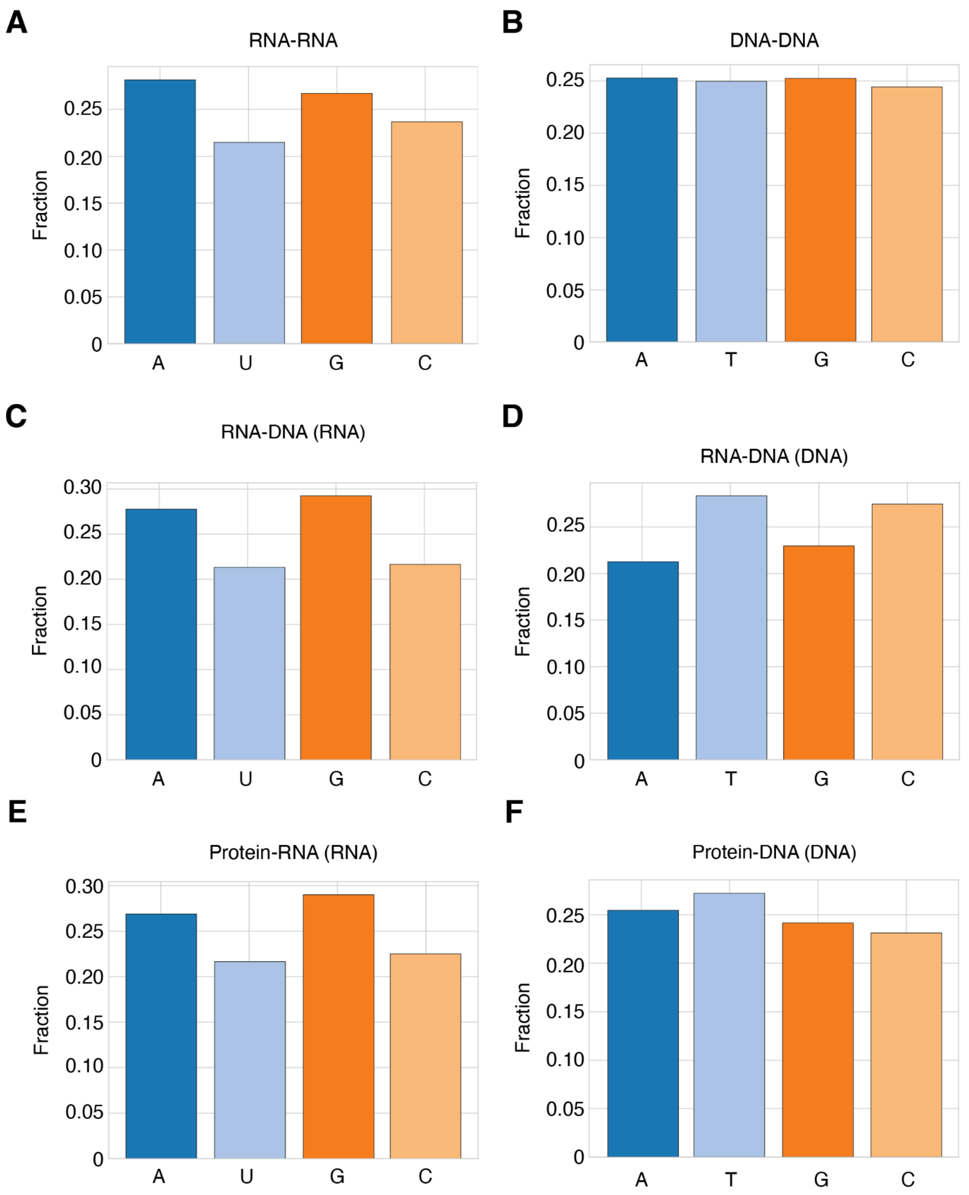

Suppl. Figure 6: Per-nucleotide type distributions (A: blue; U/T: lightblue; G: orange; C: lightorange) across nucleic acid-containing interface types. **(A)** RNA-RNA, **(B)** DNA-DNA, **(C)** RNA-DNA (RNA), **(D)** RNA-DNA (DNA), **(E)** protein-RNA (RNA), and **(F)** protein-DNA (DNA).

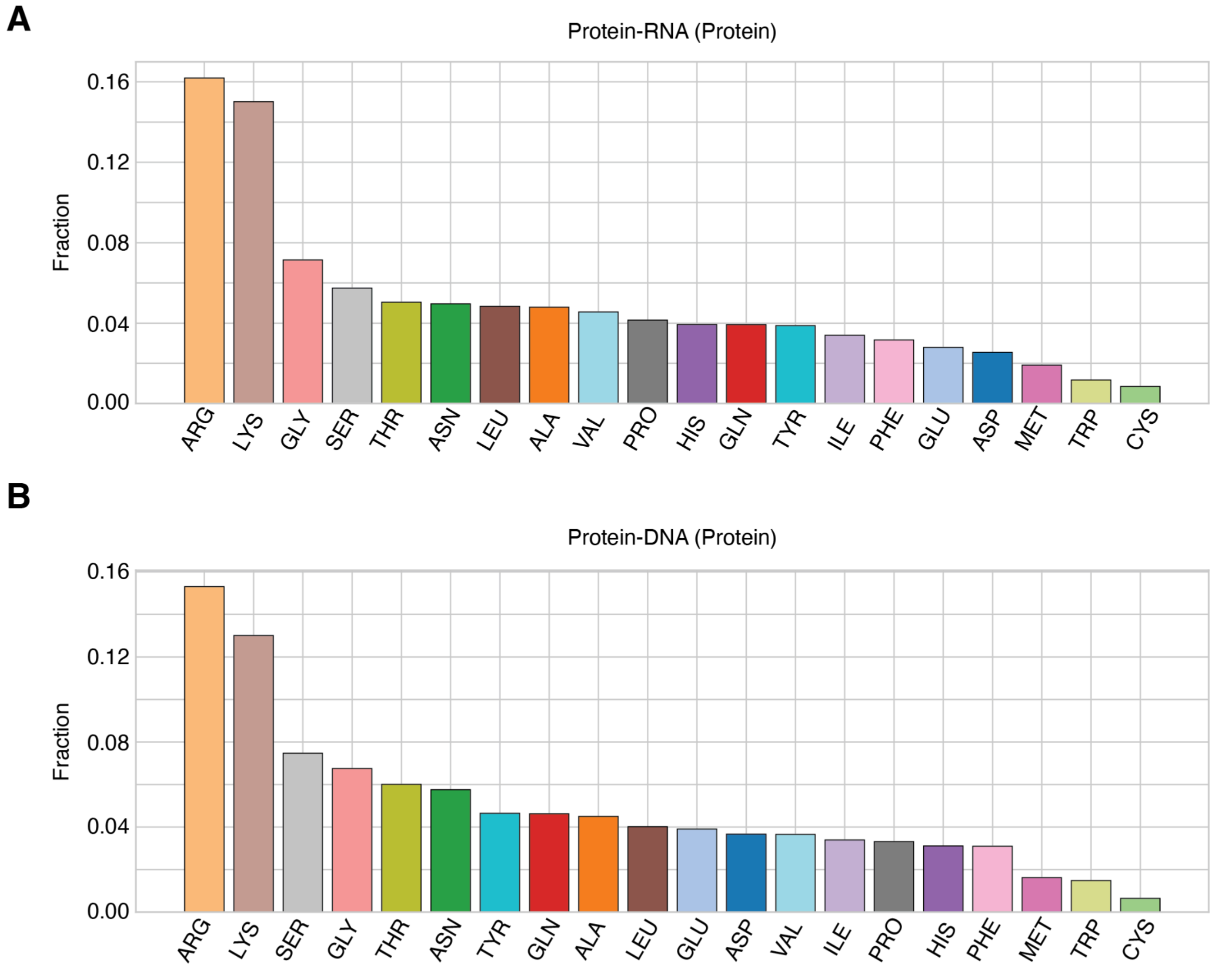

Suppl. Figure 7: Per-residue type distributions across nucleic acid-containing interface types. **(A)** protein-RNA (protein) and **(B)** protein-DNA (protein).

Supplemental tables

Suppl. Table 1: Comparison of available computational tools for structural analysis of nucleic acid-containing complexes.

Tool name	Supported molecule types	Support for predicted structures (3D)*	Confidence metric integration	cryo-EM functionality	Topology visualization	Interactive visualization	command-line support
**DNAproDB**	Protein-DNA	Yes	No	No	Yes (for DNA)	Yes	Yes
**RNAproDB**	Protein-RNA, nucleic-acid-containing complexes	Yes	No	No	Yes (for nucleic acids)	Yes	Yes
**DSSR**	Nucleic acid-containing structures	Yes	No	No	Yes (main purpose)	Yes (wDSSR web interface)	Yes
**RNAscape**	Nucleic acid-containing structures	Yes	No	No	Yes	Yes	Yes
**ViennaRNA**	RNA sequences and RNA secondary structures	No	No	No	Yes	No (static plots only)	Yes
**PPI3D**	Protein-, peptide-, and nucleic	No	No	No	No	Yes	No

	acid-containing complexes						
**PLIP**	Protein-, small-molecule-, nucleic acid-containing complexes	Yes	No	No	No	Yes	Yes
**VoroContacts**	Proteins, nucleic acids, and their complexes	Yes	No	No	No	No	Yes
**RNAView**	Nucleic acids	Yes	No	No	Yes	Yes	Yes
**ProNA3D**	Nucleic acid-containing complexes	Yes	Yes	Yes	Yes (DSSR-based)	Yes	Yes

*Support for predicted structures refers to the ability to analyze user-provided predicted 3D coordinate models (e.g., AlphaFold3), rather than the generation of predicted structures or integration of prediction confidence scores.

Suppl. Table 2: Maximum connectivity across all interface types for d_{HA} = 5 Å and d_C = 5 Å. Shown are the number of sub-interfaces analysed (count) and the distribution of maximal connectivity values, reported as median, mean, and standard deviation.

Interface type	Count	Median	Mean	Std
RNA-RNA	4,797	3.00	3.22	1.83
RNA-DNA	450	3.00	3.03	0.76
DNA-DNA	4,590	3.00	2.92	0.88
Protein-DNA	11,791	5.00	5.52	3.48
Protein-RNA	23,616	4.00	4.64	3.32

Suppl. Table 3: Mann-Whitney U (Wilcoxon rank-sum) test for maximum connectivity interface type populations.

P-value	RNA-RNA	DNA-DNA	RNA-DNA	Protein-DNA	Protein-RNA
**RNA-RNA**	x	1.18e-01	3.84e-01	<< 0.001	8.49e-110
**DNA-DNA**	x	x	1.04e-01	<< 0.001	1.87e-114
**RNA-DNA**	x	x	x	3.74e-46	1.83e-10
**Protein-DNA**	x	x	x	x	3.77e-125
**Protein-RNA**	x	x	x	x	x

Suppl. Table 4: Maximum connectivity across all interface types for d_{HA} = 5 Å and d_C = 4 Å. Shown are the number of sub-interfaces analysed (count) and the distribution of maximal connectivity values, reported as median, mean, and standard deviation.

Interface type	Count	Median	Mean	Std
RNA-RNA	5,469	3.00	3.06	1.81
RNA-DNA	460	3.00	3.00	0.78
DNA-DNA	4,685	3.00	2.91	0.89

Protein-DNA	13,792	4.00	5.06	3.47
Protein-RNA	29,264	3.00	4.26	3.21

Suppl. Table 5: Maximum connectivity across all interface types for d_{HA} = 4 Å and d_C = 5 Å. Shown are the number of sub-interfaces analysed (count) and the distribution of maximal connectivity values, reported as median, mean, and standard deviation.

Interface type	Count	Median	Mean	Std
RNA-RNA	4,954	2.00	2.67	1.35
RNA-DNA	546	3.00	2.43	0.84
DNA-DNA	4,915	3.00	2.76	0.75
Protein-DNA	13,555	3.00	3.80	2.38
Protein-RNA	31,894	3.00	3.26	2.22

Suppl. Table 6: Maximum connectivity across all interface types for d_{HA} = 4 Å and d_C = 4 Å. Shown are the number of sub-interfaces analysed (count) and the distribution of maximal connectivity values, reported as median, mean, and standard deviation.

Interface type	Count	Median	Mean	Std
RNA-RNA	6,088	2.00	2.51	1.34
RNA-DNA	781	2.00	2.21	0.85
DNA-DNA	5,832	3.00	2.64	0.81
Protein-DNA	17,375	3.00	3.39	2.31
Protein-RNA	45,631	2.00	2.97	2.07

Suppl. Table 7: Connectivity statistics by nucleotide type for DNA-DNA interfaces.

Interface type	Count	Median	Mean	Std
A	28,518	3.00	2.82	0.60

T	28,153	3.00	2.78	0.61
G	28,473	3.00	2.75	0.60
C	27,562	3.00	2.68	0.61

Suppl. Table 8: Connectivity statistics by nucleotide type for RNA-RNA interfaces.

Interface type	Count	Median	Mean	Std
A	15,874	2.00	2.44	1.31
U	12,110	2.00	2.31	1.17
G	15,056	2.00	2.34	1.17
C	13,342	2.00	2.13	1.10

Suppl. Table 9: Connectivity statistics by nucleotide type (RNA) for RNA-DNA interfaces.

Interface type	Count	Median	Mean	Std
A	1,364	3.00	2.60	0.63
U	1,047	2.00	2.31	0.68
G	1,436	3.00	2.47	0.72
C	1,063	2.00	2.39	0.63

Suppl. Table 10: Connectivity statistics by nucleotide type (DNA) for RNA-DNA interfaces.

Interface type	Count	Median	Mean	Std
A	1,014	3.00	2.64	0.66
T	1,351	3.00	2.49	0.70
G	1,095	3.00	2.64	0.62
C	1,309	2.00	2.39	0.66

Suppl. Table 11: Connectivity statistics by nucleotide type (DNA) for protein-DNA interfaces.

Interface type	Count	Median	Mean	Std

A	14,097	3.00	3.88	2.78
T	15,072	4.00	4.15	2.81
G	13,374	4.00	4.20	2.96
C	12805	3.00	3.84	2.74

Suppl. Table 12: Connectivity statistics by residue type (protein) for protein-DNA interfaces.

Interface type	Count	Median	Mean	Std
ASP	4,807	1.00	1.46	0.63
GLU	5,114	1.00	1.51	0.68
ALA	5,898	1.00	1.49	0.59
ARG	20,052	2.00	2.02	0.82
ASN	7,539	2.00	1.73	0.72
CYS	845	2.00	1.58	0.62
GLN	6,056	2.00	1.75	0.72
GLY	8,843	1.00	1.55	0.60
HIS	4,070	2.00	1.72	0.76
ILE	4,443	1.00	1.51	0.60
LEU	5,264	1.00	1.47	0.59
LYS	17,042	2.00	1.74	0.72
MET	2,123	2.00	1.66	0.68
PHE	4,061	2.00	1.63	0.63
PRO	4,337	1.00	1.51	0.59
SER	9,791	2.00	1.69	0.64
THR	7,872	2.00	1.70	0.65
TRP	1,945	2.00	1.74	0.73
TYR	6,092	2.00	1.86	0.80

VAL	4,789	1.00	1.53	0.60

Suppl. Table 13: Connectivity statistics by nucleotide type (RNA) for protein-RNA interfaces.

Interface type	Count	Median	Mean	Std
A	68,085	2.00	2.96	2.26
U	54,827	2.00	3.09	2.35
G	73,444	2.00	2.93	2.16
C	56,965	2.00	2.89	2.11

Suppl. Table 14: Connectivity statistics by residue type (protein) for protein-RNA interfaces.

Interface type	Count	Median	Mean	Std
ASP	8,397	1.00	1.75	1.04
GLU	9,224	1.00	1.76	1.01
ALA	15,824	2.00	1.95	1.12
ARG	53,411	2.00	2.87	1.62
ASN	16,357	2.00	2.21	1.21
CYS	2,806	2.00	1.87	1.15
GLN	12,961	2.00	2.27	1.32
GLY	23,561	2.00	2.08	1.07
HIS	12,987	2.00	2.58	1.48
ILE	11,211	2.00	1.86	1.09
LEU	15,944	2.00	1.85	1.01
LYS	49,536	2.00	2.53	1.41
MET	6,315	2.00	2.33	1.48
PHE	10,426	2.00	2.17	1.30
PRO	13,701	2.00	2.05	1.19

SER	18,946	2.00	2.12	1.14
THR	16,630	2.00	2.11	1.17
TRP	3,878	2.00	2.38	1.61
TYR	12,774	2.00	2.24	1.30
VAL	15,037	2.00	1.86	1.10
